# Acute Hepatitis C Outbreak among HIV-infected Men, Madrid, Spain

**DOI:** 10.3201/eid1708.110147

**Published:** 2011-08

**Authors:** Ana Montoya-Ferrer, Daniel Seth Fierer, Beatriz Alvarez-Alvarez, Miguel de Gorgolas, Manuel L. Fernandez-Guerrero

**Affiliations:** Author affiliations: Universidad Autonoma de Madrid, Madrid, Spain (A. Montoya-Ferrer, B. Alvarez-Alvarez, M. de Gorgolas, M.L. Fernandez-Guerrer);; Mount Sinai School of Medicine, New York, New York, USA (D.S. Fierer)

**Keywords:** acute hepatitis C, viruses, HIV, sexually transmitted diseases, letter

**To the Editor:** In the past decade, hepatitis C virus (HCV) has emerged as a sexually transmitted infection (STI) among HIV-infected men who have sex with men (MSM). The epidemic was originally reported in several northern European countries (England, France, Germany, and the Netherlands) (*1*) and soon after in Australia (*2*) and the United States (*3*). Acute HCV acquisition was associated with group sex, unprotected receptive anal intercourse, and according to some studies, concomitant STI (*4*). Molecular phylogenetic studies suggested evidence of an international transmission network of MSM within northern Europe (*1*). However, expansion of the HCV epidemic among MSM to Spain (*5*) or to other countries of the Mediterranean area had not previously been reported.

We report 4 cases of acute HCV in HIV-infected MSM in Madrid, Spain, 2010. These patients were monitored at a university-affiliated hospital in downtown Madrid, which provides health care to a large MSM community in the Chueca District. Diagnosis of acute HCV was made by using the following criteria of the European AIDS Treatment Network (*6*): 1) positive HCV RNA; 2) an acute rise in alanine aminotransferase level >5× the normal upper limit, with documented normal alanine aminotransferase level within 12 months; and 3) negative results for anti–hepatitis A virus immunoglobulin M and anti–hepatitis B core immunoglobulin M (when other causes of acute hepatitis were excluded). An HCV RNA load fluctuation of >1 log_10_ IU/mL, if present, was considered further evidence of acute HCV infection (*7*).

All 4 patients were MSM with well-controlled HIV infection who were receiving antiretroviral treatment. During routine medical screening, they were found to have newly elevated liver transaminase levels, and further assessment confirmed the diagnosis of acute HCV infection (Table). Three patients had received a diagnosis of STI in the previous 6 months, but only 1 patient acknowledged having unprotected anal intercourse. In addition, only 1 patient acknowledged using any recreational drugs (amyl nitrate); all denied using injection drugs (Table). All patients had lived in Madrid for at least 5 years before receiving a diagnosis of acute HCV. No patients reported having sex during international travel, using sex toys, or fisting.

**Table:** Description of 4 HIV-infected men with HCV infection, Madrid, Spain, 2010*

Characteristic	Patient 1	Patient 2	Patient 3	Patient 4
Age, y	31	41	39	39
Country of origin	Italy	Ecuador	Spain	Spain
Date of HIV diagnosis	2006 Jun	2000 Jul	1998	2001
Year ART initiated	2008	2000	2006	2006
Prior negative HCV test	2006	None†	None†	2008
Date AHC diagnosed	May 2010	May 2010	May 2010	May 2010
CD4 count, cells/μL	562	327	787	750
HIV viral load	Undetectable	Undetectable	Undetectable	Undetectable
Symptoms at diagnosis	No	Mild asthenia only	No	No
ALT/AST levels at AHC diagnosis, U/L	564/331	304/216	222/114	261/125
HCV genotype	4	4	1a	Indeterminate
HCV RNA load, IU/mL (log_10_ IU/mL)	950,556 (5.98)	629,875 (5.80)	11,827 (4.07)	2,254,258 (6.35)
HCV RNA load fluctuation within 3 mo, log_10_ IU/mL	4.32	<1.00	1.33	<1.00
Unprotected anal intercourse	No	No	No	Serosorting‡
STI in previous 6 mo	Proctitis	Syphilis	No	Proctitis
Group sex (>2 persons)	Yes	No	No	No
Drug use	Amyl nitrate only	No	No	No

*HCV, hepatitis C virus; ART: antiretroviral therapy; AHC, acute hepatitis C infection; ALT, alanine aminotransferase; AST, aspartate aminotransferase; STI, sexually transmitted infection. †Both patients had normal transaminase levels in the 4 y before AHC diagnosis. ‡Unprotected sex between seroconcordant partners (HIV positive).

The patients described here lived in the Chueca District of Madrid, the largest MSM community in Spain, which is frequented by MSM traveling from smaller cities in Spain and other countries. Two of the 3 patients were infected with HCV genotype 4, which is unusual in patients from outside the Middle East and Africa (*8*) yet unexpectedly common in northern European HCV outbreaks (*1*), which suggests that the patients reported here may have been part of the social network originating in the north. Further sequencing of these isolates is under way to address this issue. The third patient with an identifiable HCV genotype was infected with HCV genotype 1, the most common genotype among HIV-infected MSM in northern Europe (*1*). These findings suggest that a larger, undetected outbreak of HCV infection is taking place in Madrid.

Although the patients reported here described fewer risks for sexual acquisition of HCV than patients from northern Europe or the United States, 3 had recent STI, which suggests that they underreported their risks for HCV acquisition. This temporal association between STI and acute HCV in these patients suggests that the pattern of emergence of sexually transmitted HCV among MSM in Spain might be similar to that seen in northern Europe, following regional epidemics of syphilis (starting in 2000) (*9**,**10*). We therefore encourage HIV specialists and general practitioners, when investigating an STI, to perform HCV testing on MSM as well as on persons with newly elevated liver aminotransferase levels.
